# ESR Essentials: MRI-based T-staging in prostate cancer—practice recommendations by the European Society of Urogenital Radiology

**DOI:** 10.1007/s00330-026-12397-8

**Published:** 2026-02-20

**Authors:** Georgios Agrotis, Fredrik Jäderling, Amish Lakhani, Ana Sofia L. Moreira, Maka Kekelidze, Justyna Rembak-Szynkiewicz, Vibeke Logager, Harriet Thoeny, Geert Villeirs, Sungmin Woo, Ivo G. Schoots

**Affiliations:** 1 Department of Radiology, The Netherlands Cancer Institute, Amsterdam, The Netherlands; 2 GROW School for Oncology and Reproduction, Maastricht University, Maastricht, The Netherlands; 3 Department of Radiology, Capio St Göran’s Hospital, Stockholm, Sweden; 4 Institution of Molecular Medicine and Surgery (MMK), Karolinska Institutet, Stockholm, Sweden; 5 Paul Strickland Scanner Centre, Mount Vernon Cancer Centre, London, UK; 6 Department of Imaging, Charing Cross Hospital, Imperial College Healthcare NHS Trust, London, UK; 7 Department of Radiology, Unidade Local de Saúde do Algarve (ULS Algarve), Faro, Portugal; 8 Institute for Diagnostic and Interventional Radiology, University Hospital of Zurich, Zurich, Switzerland; 9 Radiology and Diagnostic Imaging Department, Maria Sklodowska-Curie National Research Institute of Oncology, Gliwice, Poland; 10 Radiological Department, Copenhagen University Hospital Herlev Gentofte, Herlev, Denmark; 11 Department of Radiology, University Teaching and Research Hospital HFR, University of Fribourg, Fribourg, Switzerland; 12 Department of Radiology, Ghent University Hospital, Ghent, Belgium; 13 Department of Radiology, NYU Grossman School of Medicine, New York, NY US; 14 Department of Radiology and Nuclear Medicine, Erasmus University Medical Center, Rotterdam, The Netherlands

**Keywords:** Prostate cancer, Magnetic resonance imaging, Neoplasm staging, Prognosis, Extraprostatic extension

## Abstract

**Abstract:**

MRI-based T-staging in prostate cancer enables more precise and individualised treatment decisions compared with traditional clinical T-staging, yet inconsistent image acquisition, interpretation, and terminology limit reproducibility and clinical integration. This report recommends standardising MRI-based T-staging to strengthen its prognostic and therapeutic relevance. The prefix “mr” for MRI-based T-staging should be used, together with “extraprostatic extension” as the official radiologic term, replacing “extracapsular extension”, to ensure consistency with pathology and the UICC TNM system. Within mrT3a disease, radiologists should differentiate “focal” from “established” extraprostatic extension, as established disease carries a higher recurrence risk and often requires more extensive surgical or radiotherapeutic management. Quality assurance programs, standardised acquisition protocols, and targeted education for radiologists and technologists are essential for improving interinstitutional consistency. Clinicians should incorporate proposed terminology and focal/established subclassification into structured reports and multidisciplinary discussions to refine risk stratification and guide nerve-sparing surgery or adjuvant therapy decisions. These coordinated measures will enhance the reproducibility and prognostic value of MRI-based T-staging, reduce overtreatment, and improve patient outcomes in contemporary prostate cancer management.

**Key Points:**

- *The prefix “mr” should be added to T-staging for reporting and multidisciplinary team guidance, distinguishing it from clinical and pathological T-staging; extraprostatic extension should be explicitly defined as mrT3a.*

- *Staging assessment should be refined by subclassifying focal versus established extraprostatic extension to better guide risk stratification and treatment decisions.*
- *More reproducible and objective criteria of MRI-derived parameters should be adopted to reduce interpretation variability and to improve reader reproducibility.*

## Key recommendations

- Standardise MRI T-staging terminology: Adopt the prefix “mr” (e.g., mrT2, mrT3a) to clearly distinguish imaging-based from clinical and pathological staging. Use “extraprostatic extension” instead of ambiguous terms like “extracapsular extension” to improve cross-disciplinary communication and clinical integration (Evidence level: moderate).
- Refine MRI T-staging assessment: Increase the use of more objective MRI-derived parameters, and incorporate subclassification of focal versus established extraprostatic extension, reflecting pathological consensus, and enhancing treatment planning and prognostication (Evidence level: moderate).
- Ensure quality and training programs in MRI T-staging: Harmonise MRI acquisition protocols, apply standardised MRI quality tools, and implement structured reader training to ensure consistent diagnostic accuracy. Implement certification or benchmarking across centres to improve interpretation and reader consistency (Evidence level: moderate).

## Introduction

Prostate cancer remains one of the most commonly diagnosed malignancies in men, characterised by significant heterogeneity in biological behaviour, disease stage, and clinical long-term outcomes. Pathologically locally advanced disease (pT-stage 3–4) has a worse prognosis compared with organ-confined disease (pT-stage 1–2), and cancers with seminal vesicle invasion (pT3b) show worse outcomes compared with cancers extending beyond the prostate pseudocapsule (pT3a) [1]. Furthermore, pathologically assessed established extraprostatic disease carries worse prognostic significance than focal extraprostatic disease, both categorised as pT3a.

MRI assists in clinical T-staging, as recommended by international guidelines, to improve concordance with pathology outcomes [2]. The important distinction in prognostic outcome between T-stages highlights the need for accurate MRI-based T-staging, also differentiating between established and focal extraprostatic extension, to further improve preoperative risk stratification and subsequent treatment decision.

The current recommendations on MRI-based T-staging, provided by expert members of the European Society of Urogenital Radiology (ESUR), signal the urgent need for consensus-driven standardisation, proposing future directions to further improve clinical decision-making and patient outcomes. An approach more focused on specificity by lowering false positive results may reduce overstaging and the associated escalated treatment harm [3].

## MRI T-staging: reporting, separate from clinical T-staging

Men with clinically organ-confined disease typically receive less aggressive therapeutic interventions than men with locally advanced disease. International guidelines predominantly rely on clinical T-staging determined by digital rectal examination within established prognostic frameworks, such as the D’Amico risk classification or Cambridge Prognostic Grouping. However, MRI provides superior anatomical detail of the tumour and its local extension. Consequently, MRI-based T-staging plays a crucial role in guiding treatment decisions, which vary substantially between prostate cancer T-stages. Therefore, the European Association of Urology (EAU) prostate cancer guidelines recommend reporting MRI-based T-stage separately from clinical T-stage [2, 4].

MRI-based T-staging can aid in the stratification from low- to high-risk categories, directly influencing therapeutic strategy and postoperative management in men with prostate cancer. For instance, in high-risk men, radiation treatment may require extended-field treatment involving lymph nodes in combination with prolonged androgen deprivation treatment (e.g., 2–3 years) to maximise cancer control. In high-risk surgical candidates, radical prostatectomy typically involves non-nerve-sparing procedures and is frequently complemented by extended pelvic lymph node dissection, aiming to realise comprehensive oncological clearance, at the expense of increased risks of postoperative morbidity.

To minimise ambiguity across radiology, pathology, and clinical assessments, ESUR proposes to adopt the prefix ‘mr’ to T-stage when referring to MRI-based T-staging. This allows a clear distinction from digital rectal examination-based clinical T-staging and pathological T-staging (Table 1). Extraprostatic extension explicitly refers to mrT3a disease only, mirroring pathology adopted terminology as well as the TNM 9th edition update [5, 6], therefore neither including seminal vesicle invasion (mrT3b), nor invasion into adjacent organs (mrT4).

**Table 1:** Proposed MRI-based (mr) T-staging terminology in men with prostate cancer

Stage	Definition
mrT2	Organ-confined disease, no imaging evidence of extraprostatic extension.
mrT3a	Imaging evidence of extraprostatic extension, which means tumour extending beyond the border or pseudocapsule of the prostate into periprostatic tissues. This definition should be restricted to extension beyond the prostate pseudocapsule (posterior, posterolateral, apex, bladder neck, anterior fibromuscular stroma, neurovascular bundles, sphincter complex, pelvic floor) but excluding the seminal vesicle invasion. Subclassification into “focal” versus “established” mrT3a disease is proposed, in analogy to pathology (pT3a), to reflect prognostic significance.
mrT3b	Imaging evidence of seminal vesicle invasion, which must be reported separately, given its distinct prognostic implications.

The prefix ‘mr’ distinguishes from the prefix ‘i’ (i.e., “imaging”) and the prefix ‘mi’ (i.e., “molecular imaging”), which are increasingly used in international reports on TNM staging by the imaging modality of prostate-specific membrane antigen positron emission tomography and computed tomography (PSMA PET-CT) [7]. Subsequently, the prefix ‘mr’ in T-staging may avoid confusion when ‘mi’ is used in TNM staging.

## MRI T-staging: variable terminology of extraprostatic extension

In prostate cancer, the description of tumour spread beyond the gland has historically varied across disciplines, resulting in inconsistent T-staging terminology. Terms such as extracapsular extension, extracapsular breach, capsular invasion or capsular penetration may have similar meaning; however, they can be interpreted differently.

**Fig. 1:**
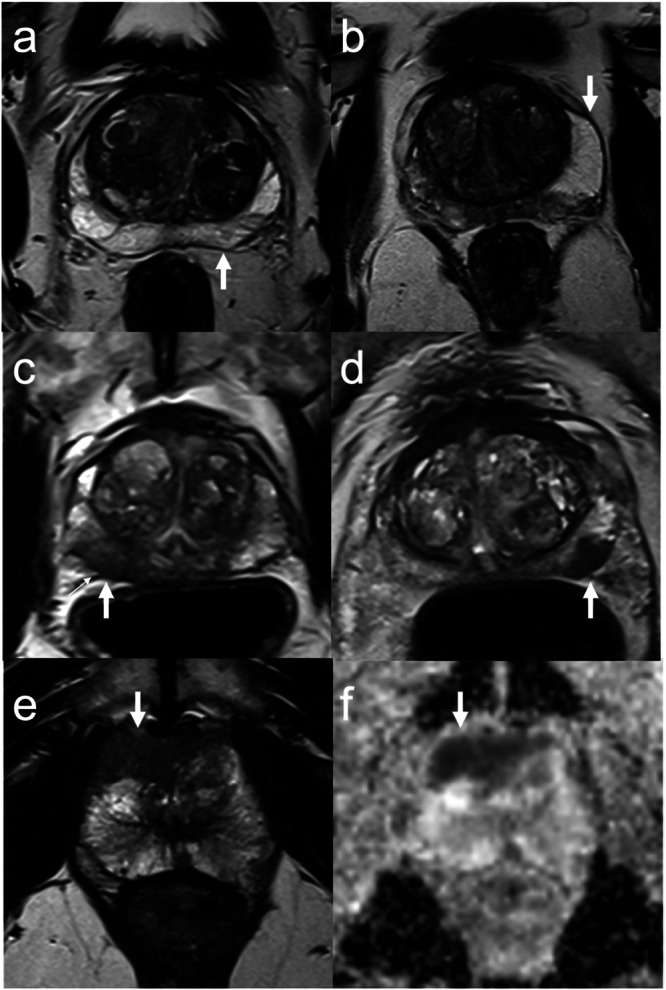
MRI-derived parameters for extraprostatic extension prediction in MRI T-staging. A low-signal-intensity band on T2-weighted images represents the so-called prostate “pseudocapsule”. This pseudocapsule is more distinct at the dorsal side of the peripheral zone (arrow) (**a**) than at the ventral side of the transition zone (arrow) (**b**). Irregularity (arrow) (**c**) and bulging (arrow) (**d**) of the pseudocapsule due to likely cancerous tissue in the dorsal peripheral zone can be assessed on high-quality T2-weighted images. In MRI T-staging, this focal breach of either mrT3a or mrT2 disease is challenging to correctly predict focal pT3a or pT2a. Irregularity of the pseudocapsule may be assessed as focal extraprostatic extension (focal mrT3a), while bulging of the pseudocapsule may be assessed as organ-confined disease (mrT2), despite the higher risk of focal extraprostatic extension at pathology (focal pT3a). Clear disruption of the pseudocapsule at the anterior border of the transition zone, combined with frank extracapsular breach (arrow) on T2w-images (**e**) and diffusion weighted images (**f**), should be assessed as established extraprostatic extension (established mrT3a). Pathology assessment showed established pT3a

In radiology reading assessments, the term “extracapsular” extension is often used to describe MRI findings suggestive of tumour spread beyond the prostate into the periprostatic connective tissue. This reflects the radiological focus on disruption of the low-signal-intensity band on T2-weighted images representing the so-called prostate “capsule” (Fig. 1a, b). However, MRI features such as pseudocapsule irregularity or loss do not confirm pathological extraprostatic extension (Fig. 1c, d). Histologically, a true capsule does not exist; rather, it consists of concentrically arranged fibromuscular tissue continuous with the prostatic stroma. Its outer surface forms fibromuscular bundles that merge into the periprostatic connective tissue, while at the apex, where glandular elements are sparse, this fibromuscular layer is no longer present [8].

**Fig. 2:**
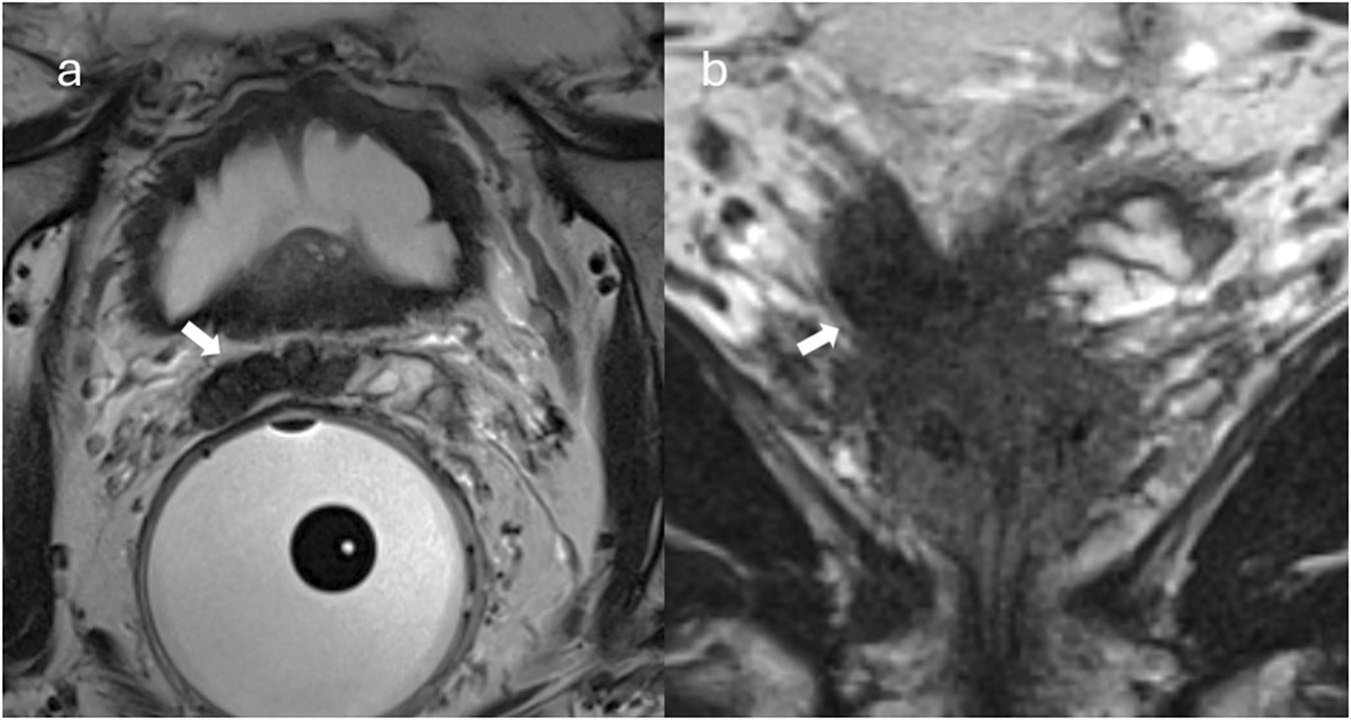
MRI-derived parameter for seminal vesicle invasion in MRI T-staging. Axial (**a**) and coronal (**b**) T2-weighted MR images demonstrating seminal vesicle invasion (SVI). MRI assessment of SVI relies on identifying direct tumour extension from the prostate base into the seminal vesicle (arrow), loss of the normal low-signal-intensity wall and replacement of the seminal vesicle lumen by intermediate to low tumour signal

In pathology reading assessments, ‘extraprostatic extension’ is now the official internationally adopted term, defined as histological evidence of tumour extending beyond the confines of the prostate into the periprostatic soft tissue, endorsed by the International Society of Urological Pathology (ISUP), also distinguishing “extraprostatic extension” (pT3a) from “seminal vesicle invasion” (pT3b) (Fig. 2) [5].

However, confusion has arisen because the term “extraprostatic extension” is sometimes used interchangeably with “extracapsular extension” to describe T3a disease and, in some reports, even used to include seminal vesicle invasion (T3b). Although both reflect tumour spread beyond the prostate, seminal vesicle invasion carries a distinctly worse prognosis. The recent updated UICC TNM classification for prostate cancer (9th edition) has also standardised the terminology to “extraprostatic extension”, replacing “extracapsular extension” [6].

This inconsistent use of terminology between radiologic and pathologic staging complicates interpretation, particularly when MRI-based staging demonstrates independent prognostic value for outcomes such as biochemical recurrence and metastasis [9, 10]. Therefore, the ESUR advocates for explicitly adopting “extraprostatic extension” as the official term in radiologic assessment. In MRI-based staging, “extraprostatic extension” (mrT3a) refers solely to tumour extension beyond the prostate capsule and does not include seminal vesicle invasion (mrT3b) or invasion of adjacent organs (mrT4) such as the bladder, rectum, sphincter, or pelvic wall.

## MRI T-staging: variable terminology of “focal” and “established” extraprostatic extension

The ISUP and International Collaboration on Cancer Reporting (ICCR) both have advocated for subclassifying locally advanced disease into ‘focal’ and ‘established’ extraprostatic extension, given the prognostic relevance and potential impact on treatment decisions [5, 11]. Unfortunately, no consensus has been reached on the precise histological definition of focal extraprostatic extension or on the methods for quantitatively assessing its extent. This also highlights the variability in practices in pathology reporting [12].

Radiological societies urge for consensus on MRI T-staging terminology; however, unifying nomenclature is challenging given the inter-reader variability among radiologists and pathologists in identifying extraprostatic extension. In addition, the distinction between focal versus established, microscopic versus macroscopic, minimal versus extensive, or non-visible versus visible extension has been variably applied in the radiological and pathological literature. Importantly, the pathological definitions of focal versus established extension do not directly translate to radiology. In pathology, “focal” extension refers to only one or a few neoplastic cells extending beyond the prostatic “capsule”, whereas in radiology, “focal” features such as capsular irregularity or abutment are expected to be associated with more favourable outcomes compared to measurable or established extraprostatic extension, similarly to pathology findings [1]. Clarifying how the prognostically favourable “focal” category in pathology corresponds to specific imaging features is essential to ensure consistent interpretation and prognostic alignment between the two disciplines.

This may improve preoperative risk stratification, ultimately adopting prognostic MRI-derived staging parameters into risk-group modelling and into the official TNM for prostate cancer staging.

## MRI T-staging: variable direct and indirect parameters

**Fig. 3:**
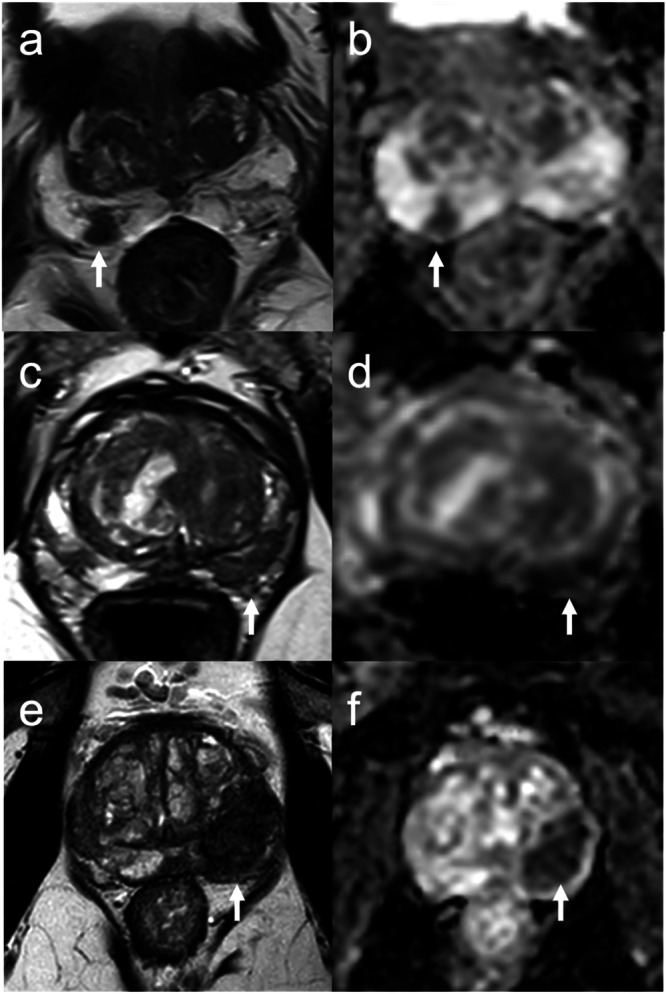
Treatment outcomes of men with prostate cancer, following MRI T-staging. A 67-year-old male with mrT2 disease (organ-confined) in the right peripheral zone (arrow) on T2-weighted imaging (**a**) and apparent diffusion coefficient (ADC) (**b**), underwent radical prostatectomy. High-grade cancer (Grade Group 3) was confirmed. Pathology showed pT2 disease. Biochemical recurrence was not observed in 64 months of follow-up. A 62-year-old male with minimal locally advanced disease (focal mrT3a), in the left peripheral zone (arrow) with pseudocapsule abutment and pseudocapsule irregularity, with tumour pseudocapsule contact length of 18 mm, on T2W-imaging (**c**) and ADC (**d**), scored as PI-RADS 5, underwent radical prostatectomy with unilateral nerve-sparing surgery. High-grade cancer (Grade Group 4) was confirmed. Pathology showed focal pT3a disease without positive surgical resection margins. Biochemical recurrence was not observed in 28 months of follow-up. A 74-year-old male with convincing locally advanced disease (established mrT3a) on T2w-imaging (**e**) and ADC (**f**), with established extraprostatic extension (arrow) including mesorectal fascia involvement, was scored as PI-RADS 5. High-grade cancer (Grade Group 4) was confirmed by targeted biopsies. This man underwent radiation treatment with prolonged androgen deprivation treatment. Biochemical recurrence occurred after 14 months of follow-up

MRI-based identification of extraprostatic extension relies on direct and indirect signs. Measurable tumour extension beyond the prostatic tissue into the periprostatic fat is a direct sign of extraprostatic extension and carries a strong positive predictive value [3]. Histological studies further demonstrate that the radial extent of extraprostatic extension is positively correlated with recurrence after radical prostatectomy (Fig. 3) [13].

Indirect signs of extraprostatic extension consist of tumour pseudocapsule contact length, pseudocapsular bulging, neurovascular bundle thickening, recto-prostatic angle obliteration, and modified capsular configuration such as thickening, disruption, irregularities, or spiculations over the tumour surface [14]. Other indirect signs are low apparent diffusion coefficient (ADC) values and larger tumour volume of lesions in the peripheral zone. However, most of these indirect signs are subjective and suffer from reduced inter-reader reproducibility, resulting in inconsistent MRI T-staging and limited clinical utility.

Nevertheless, reporting the risk of extraprostatic extension at these sites is important for surgical planning, particularly when considering nerve-, urethral-, and bladder neck-sparing procedures. Anterior prostate cancers also differ from posterior tumours, showing larger volumes, lower Gleason (ISUP) grading, and fewer cases of extraprostatic extension, but higher rates of positive surgical margins [15].

## MRI T-staging: variable diagnostic accuracy

Two recent meta-analyses on MRI-based T-staging demonstrated high specificity (0.87 [0.82–0.91] and 0.91 [0.88–0.93]) but limited sensitivity (0.55 [0.43–0.66] and 0.57 [0.49–0.64]) for detecting extraprostatic extension. Both analyses showed comparable diagnostic performance between biparametric (bpMRI) and multiparametric MRI (mpMRI), with mpMRI showing only marginally higher sensitivity but equivalent or slightly lower specificity. Overall, mpMRI did not demonstrate a significant performance advantage over T2-weighted imaging alone or in combination with functional sequences. This indicates that EPE assessment can be reliably performed using either bpMRI or mpMRI protocols [16, 17].

Over the past three decades, numerous approaches have been explored to improve the accuracy of MRI-based extraprostatic extension. The radiological-pathological discordance in determining organ-confined and locally advanced disease is largely attributed to MRI’s inability to reliably detect microscopic extraprostatic extension. Striving to detect every case of extraprostatic extension with MRI inevitably leads to false positive results and the risk of subsequent overtreatment [18].

Overall, the diagnostic accuracy among MRI T-staging studies in surgically treated men with prostate cancer varies widely, owing to variable MRI protocols and acquisitions, heterogeneous quality of studies, and diverse MRI equipment. The wide variation in diagnostic metrics may also be related to differences in scoring systems for MRI T-staging [3].

## MRI T-staging: variable prognostic value

The prognostic significance of pathological locally advanced disease is variable and depends on its tumour extent [1, 12, 13]. Specifically, prostate cancers with pathologically “established” extraprostatic extension have a significantly worse prognosis than cancers with only “focal” disease [19]. Cancers with such established extraprostatic extension have a higher risk of positive surgical resection margins and biochemical recurrence compared to cancers with focal extraprostatic disease, while both groups are currently staged as pT3a.

In accordance with the variable oncological outcomes of focal and established pT3a disease, increasing evidence suggests that the prognostic significance of radiological locally advanced disease (mrT3) is also variable [20]. Men with established mrT3a and pT3a disease at radical prostatectomy have much worse long-term oncological outcomes than men with focal mrT3a but pT2 disease. Strikingly, men with mrT3a but pT2 cancers on radical prostatectomy have similar long-term oncological outcomes as compared to men with mrT2 but focal pT3a cancers on radical prostatectomy [9]. This suggests that indirect signs of extraprostatic extension, such as pseudocapsule irregularity or pseudocapsule loss, proposed as focal mrT3 but locally advanced disease, may result in unnecessarily escalated treatment regimen [9, 21, 22]. Ongoing research efforts aim to align the radiologic definitions of focal and established extraprostatic extension with their pathological counterparts and respective prognostic groups, to better determine how individual imaging signs should be weighted and prioritised in future standardised scoring systems.

## MRI T-staging: variable reader reproducibility

MRI-based identification of extraprostatic extension is highly variable and depends on the radiologist’s expertise. Expertise is mainly based on pathological feedback, compounded by the subjective nature of assessment and the plethora of different descriptors and staging definitions used by various authors to date [3, 14]. Experienced readers showed significantly higher accuracy in assessment of extraprostatic extension than less experienced readers (AUC 0.86 [0.79–1.00] vs. 0.65 [0.56–0.82] [109]) and 0.90 [0.79–1.00] vs. 0.69 [0.56–0.82] [23, 24].

Extraprostatic extension is most readily identified histologically at the posterior and posterolateral prostate, where tumour cells infiltrate periprostatic fat. In contrast, MRI T-staging becomes more challenging when tumours abut the anterior rectal wall, pelvic floor or bladder neck. Assessment at the apex is particularly difficult due to the lack of distinct anatomical boundaries on both MRI and histology (Fig. 1e, f).

To minimise inter-reader variability and improve staging accuracy, structured training programs and certification initiatives are increasingly emphasised. Dedicated hands-on workshops and focused didactic sessions have been shown to enhance interpretive skills. For example, a training intervention increased extraprostatic extension accuracy in eleven radiological fellows (from 0.50 [0.35–0.70] to 0.81 [0.63–0.96]) [25]. The ESUR, as part of the ESR European Training Curriculum, offers a biannual “European Certification in Prostate MRI” which ensures a broad baseline proficiency covering prostate MRI-scoring systems and EAU prostate cancer guideline-based indications. Such structured educational frameworks are likely to promote minimal competency standards, improve inter-reader agreement, and ultimately enhance the reliability of MRI T-staging in clinical practice.

## MRI T-staging: variable image quality

The accuracy of MRI T-staging in prostate cancer, particularly in detecting extraprostatic extension, is strongly dependent on image quality. Despite the widespread adoption of PI-RADS-compliant MRI protocols, the quality of the MRI acquisition remains heterogeneous across institutions, prompting the development of the Prostate Imaging Quality (PI-QUAL) score to standardise quality assessment [26].

Higher-quality scans are associated with significantly improved cancer detection and more accurate T-staging, while low-quality scans increase the risk of pathological upstaging and reduce diagnostic confidence in extraprostatic extension evaluation [27]. Post-biopsy changes can further complicate interpretation, underscoring the need to review pre-biopsy imaging or repeat MRI when necessary. Structured quality improvement programs, including protocol harmonisation, technologist training, and ongoing quality control, have been shown to increase optimal-quality imaging and cancer detection rates [28].

Artificial intelligence-based quality assessment has also been shown to significantly improve the accuracy of detecting extraprostatic extension. This is particularly relevant because poor-quality MRI scans can reduce radiologist performance by more than 20% [29].

Rigorous image quality assurance, whether through PI-QUAL scoring, structured training, or automated artificial intelligence-based quality tools, is essential to unlock the full diagnostic and prognostic potential of MRI in prostate cancer.

## MRI T-staging: the need for a universal scoring system

**Fig. 4:**
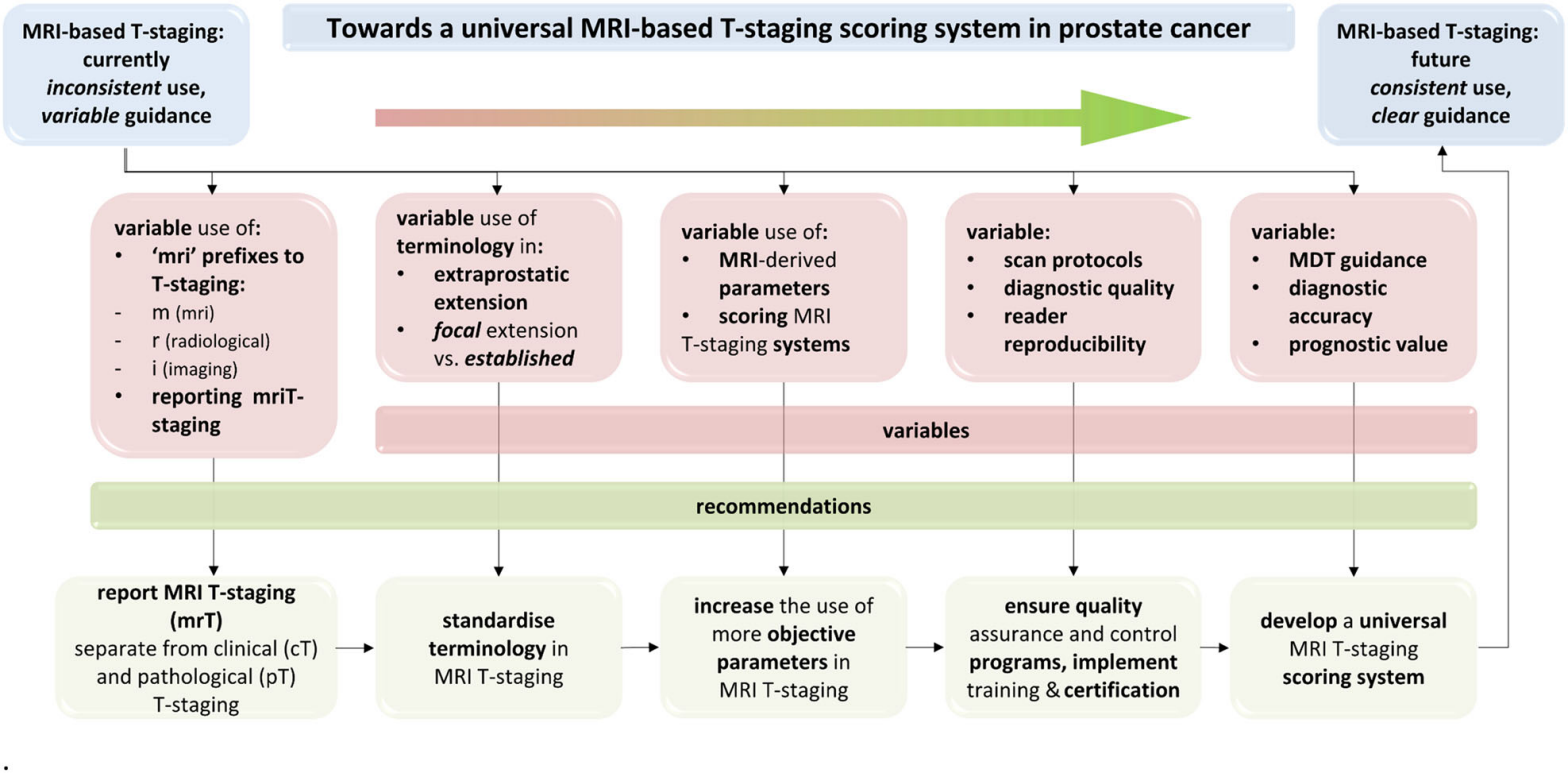
MRI T-staging: practice recommendations towards a universal scoring system in prostate cancer. Variable MRI parameters currently result in inconsistent MRI T-staging with reduced guidance in multidisciplinary meetings for treatment decisions and prognostication (blue box, upper left). Ultimately, clear guidance in multidisciplinary meetings could be realised by consistent use of a universal MRI T-staging scoring system (blue box, upper right), overcoming variables (red) by systematically adopting practice recommendations (green)

An approach towards a refined MRI T-staging scoring system for extraprostatic extension, built on reproducible and objective imaging features, would provide greater consistency while also strengthening the role of MRI in accurate staging and clinical decision-making. In addition, standardised radiological terminology should be adopted, and MRI-derived criteria for extraprostatic extension should be incorporated [3]. Crucially, such a system should also distinguish between direct and indirect signs of extraprostatic extension, to balance predictive value with clinical relevance. Furthermore, radiological terminology should be aligned with the pathological terminology regarding ‘focal’ and ‘established’ locally advanced disease. A refined MRI T-staging system would enhance risk stratification and enable better-informed treatment decisions, ultimately improving quality of life without compromising long-term oncological outcomes. To ensure global consistency, a proposal for universal terminology and standardisation should be developed through international radiological societies in close coordination with ISUP and ICCR (Fig. 4).

To achieve a more clinically appropriate MRI-based T-staging system, a consensus meeting between radiologists and pathologists, in collaboration with urologists, radiation and medical oncologists, is envisioned to update the ESUR guidelines for MRI-based extraprostatic extension prediction. Discussions within the ESUR are already moving toward such a multidisciplinary initiative, aiming to harmonise radiologic and pathologic perspectives. Furthermore, multicentre prospective studies will be essential to validate any proposed system and to generate the level of evidence required for guideline integration.

## Summary statement

The introduction of MRI T-staging has fundamentally shifted the paradigm in prostate cancer management, demonstrably outperforming current clinical T-staging by facilitating individualised treatment decisions and optimising the balance between oncological control and quality-of-life preservation. However, the full diagnostic and prognostic potential of MRI remains unrealised, hampered by widespread variability in image acquisition, interpretation heterogeneity and inconsistency in terminology. This current lack of standardisation limits inter-reader reproducibility and restricts the broader integration of MRI-derived parameters into clinical risk grouping frameworks.

To take the next step of MRI T-staging in prostate cancer, the radiological community must embrace an urgent, consensus-driven standardisation effort. This requires harmonisation of terminology, specifically adopting the distinction between focal and established extraprostatic extension within locally advanced disease. This also necessitates robust quality assurance programs, the adoption of standardised protocols, structured education to promote minimal competency standards, and the validation of a refined MRI-scoring system built on reproducible imaging features (Table 2). By unifying methodology, the radiological community is able to strengthen risk stratification, enhance prognostic value, and finally ensure that MRI T-staging fulfils its promise as a robust, consistent, and indispensable tool for improving patient outcomes worldwide.

**Table 2:** Practice recommendations towards a universal MRI T-staging scoring system by the European Society of Urogenital Radiology

No.	Practice recommendations
1	Report MRI-based T-staging separately from clinical and pathological T-staging to highlight MRI’s contribution to individualised treatment planning and prognostic assessment in prostate cancer management.
2	Use “extraprostatic extension” (EPE) in MRI T-staging as the standard term in radiological reporting to ensure alignment with pathological terminology and to improve interdisciplinary communication.
3	Standardise the definitions of “focal” and “established” EPE in MRI T-staging, in coordination with international radiological and pathological societies, to enhance consistency and risk stratification.
4	Differentiate between “focal” and “established” EPE in MRI T-staging to reflect prognostic differences and prevent overtreatment of subtle or indirect findings.
5	Reduce reliance on indirect and subjective MRI features by adopting more reproducible and objective criteria to minimise variability in MRI T-staging.
6	Ensure quality assurance and quality control programs, harmonising MRI acquisition protocols, hardware standards, image quality, and assessment systems across centres, to reduce variability in MRI T-staging diagnostic accuracy.
7	Implement structured education, training, and certification programs to establish minimal competency standards and improve inter-reader agreement in MRI T-staging.
8	Develop and validate a universal refined and standardised MRI-based scoring system for prostate cancer T-staging, based on objective and reproducible imaging features to enhance communication, risk stratification, and treatment decision-making.

## Patient summary

MRI is an essential tool for staging prostate cancer, helping doctors see whether the disease has spread outside the gland or is still confined. Accurate staging is crucial, as it directly influences treatment choices such as surgery, radiotherapy or hormonal treatment. This paper summarises recommendations to standardise MRI-based T-staging terminology, ensuring clearer communication between radiologists, urologists, and pathologists. It also proposes refining MRI-based T-staging by distinguishing between limited (“focal”) and more extensive (“established”) tumour spread, which can impact patients’ treatment and outcome. Finally, we highlight the importance of image quality and structured radiologist training to reduce errors and improve patient care.

## Abbreviations

ADC: Apparent diffusion coefficient
PI-RADS: Prostate Imaging Reporting and Data System
PSMA: Prostate-specific membrane antigen
